# Severe Persistent Neonatal Immune Thrombocytopenia Despite Optimized Maternal Chronic Immune Thrombocytopenia (ITP) Management

**DOI:** 10.7759/cureus.102833

**Published:** 2026-02-02

**Authors:** Nick Trevino, Gabriella Kuffour, Adora Okogbule-Wonodi, Shannon Wentworth, Ahmed Ali, Mekdem Bisrat, Miriam Michael, Inez Reeves

**Affiliations:** 1 Pediatrics and Child Health, Howard University College of Medicine, Washington DC, USA; 2 Pediatrics and Child Health, Howard University Hospital, Washington DC, USA; 3 Hematology and Medical Oncology, Howard University Hospital, Washington DC, USA; 4 Internal Medicine, Howard University Hospital, Washington DC, USA

**Keywords:** maternal antiplatelet antibodies, maternal–fetal immunology, neonatal thrombocytopenia, platelet transfusions, pregnancy complications

## Abstract

Immune thrombocytopenia (ITP) is the most common cause of thrombocytopenia in pregnancy, with maternal autoantibodies crossing the placenta and predisposing neonates to severe thrombocytopenia. Neonatal immune thrombocytopenia (NIT) can result in significant morbidity, including intracranial hemorrhage. We report a term neonate born to a mother with chronic severe ITP. Despite antenatal therapy with corticosteroids, intravenous immunoglobulin (IVIG), and elective cesarean delivery at a safe maternal platelet threshold, the neonate presented with severe thrombocytopenia requiring multiple platelet transfusions, repeated courses of IVIG, and adjunctive steroids. The infant’s course was prolonged, requiring 29 days of hospitalization, though ultimately discharged with recovery of platelet counts. This case underscores the persistent risk of NIT despite optimal maternal management, highlights maternal predictors such as prior affected offspring and history of splenectomy, and illustrates the therapeutic dilemmas in neonatal care. Early recognition and coordinated multidisciplinary management remain critical to reducing morbidity in mother-infant dyads affected by ITP.

## Introduction

Immune thrombocytopenia (ITP) is an autoimmune disorder characterized by immune-mediated platelet destruction and impaired platelet production, resulting in thrombocytopenia, defined as a platelet count <150 × 10⁹/L [1]. It is the most common cause of maternal thrombocytopenia in pregnancy, with an incidence estimated between 1 in 1,000 and 1 in 10,000 pregnancies, nearly 10-fold higher than in the general population [2,3]. While many pregnant patients remain asymptomatic, severe maternal thrombocytopenia can increase risks for peripartum hemorrhage, complicate delivery planning, and necessitate treatment with corticosteroids, intravenous immunoglobulin (IVIG), or platelet transfusions [4,5].

Beyond maternal morbidity, ITP has significant implications for the neonate. Maternal IgG autoantibodies cross the placenta and bind fetal platelet glycoproteins (GPIIb/IIIa, GPIb/IX), leading to neonatal immune thrombocytopenia (NIT) [6]. Clinical manifestations range from petechiae and bruising to mucosal bleeding and, in rare cases, life-threatening intracranial hemorrhage [7,8]. Studies show that severe maternal disease, a history of splenectomy, and a prior affected infant are among the strongest predictors of neonatal thrombocytopenia [9-11].

Despite recognition of these risks, predicting neonatal outcomes remains challenging, and there are no universally accepted guidelines tailored specifically to the mother-infant dyad [12]. Here, we describe a case of chronic maternal ITP resulting in severe, persistent NIT requiring multimodal therapy. This case underscores the limitations of current approaches, highlights maternal and neonatal predictors of disease severity, and illustrates the need for multidisciplinary guidelines to optimize care.

## Case presentation

Maternal history

**Figure 1:**
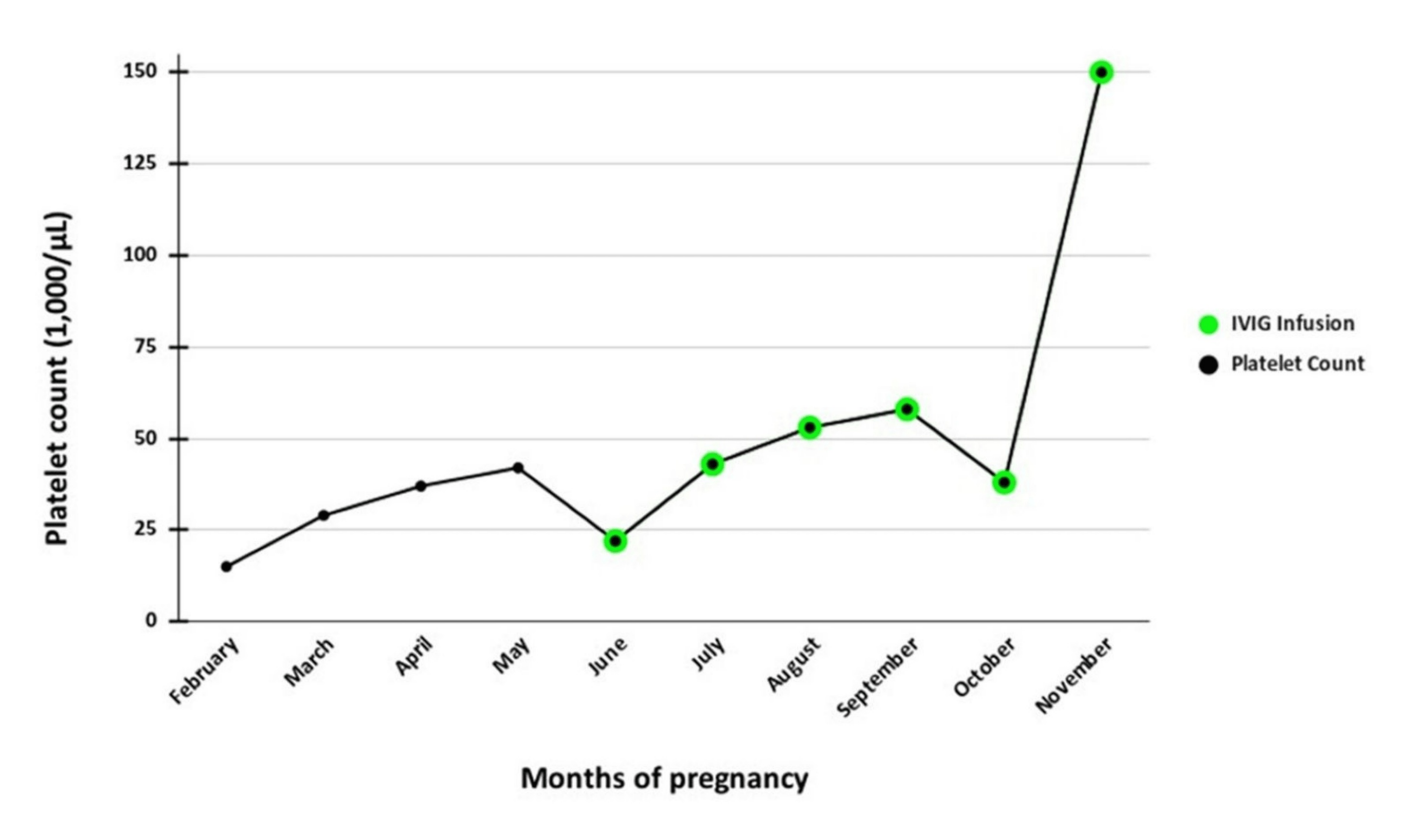
Maternal platelet counts and intravenous immunoglobulin (IVIG) treatment during pregnancy.

A 29-year-old G2P1001 Hispanic female with childhood-diagnosed chronic ITP presented at 39 weeks of gestation for elective repeat cesarean delivery. She underwent splenectomy at nine years of age in 2006. Despite prior splenectomy, her platelet count ranged from 17,000/µL to 81,000/µL throughout the pregnancy, and her pre-pregnancy measured platelet count ranged from 15,000/µL to 20,000/µL. During pregnancy, 40 mg of dexamethasone was given for four days, but they were unable to bring it to the target platelet count. Intermittent courses of prednisone were prescribed prior to pregnancy, but she was not always compliant with taking them. Subsequently, she received a prolonged prednisone taper and intravenous immunoglobulin (IVIG) infusions, with platelet counts targeted at >50,000/µL before delivery (Figure 1). At delivery, her platelet count was 150,000/µL. Her first pregnancy had been complicated by severe neonatal thrombocytopenia (nadir 10,000/µL).

Infant history

A term female neonate (birth weight 3,290 g) was delivered with Apgar scores of 9 and 9 at one and five minutes, respectively. Physical examination revealed petechiae, ecchymoses, and a right upper extremity hemangioma. Admission platelet count was 18,000/µL. She received two platelet transfusions and a three-day IVIG course, but thrombocytopenia persisted. For delivery, neuraxial (spinal) anesthesia was used, and the estimated blood loss was around 700 mL. The maternal platelet count prior to delivery was 41,000/µL, and she received no platelet transfusion after delivery.

**Figure 2:**
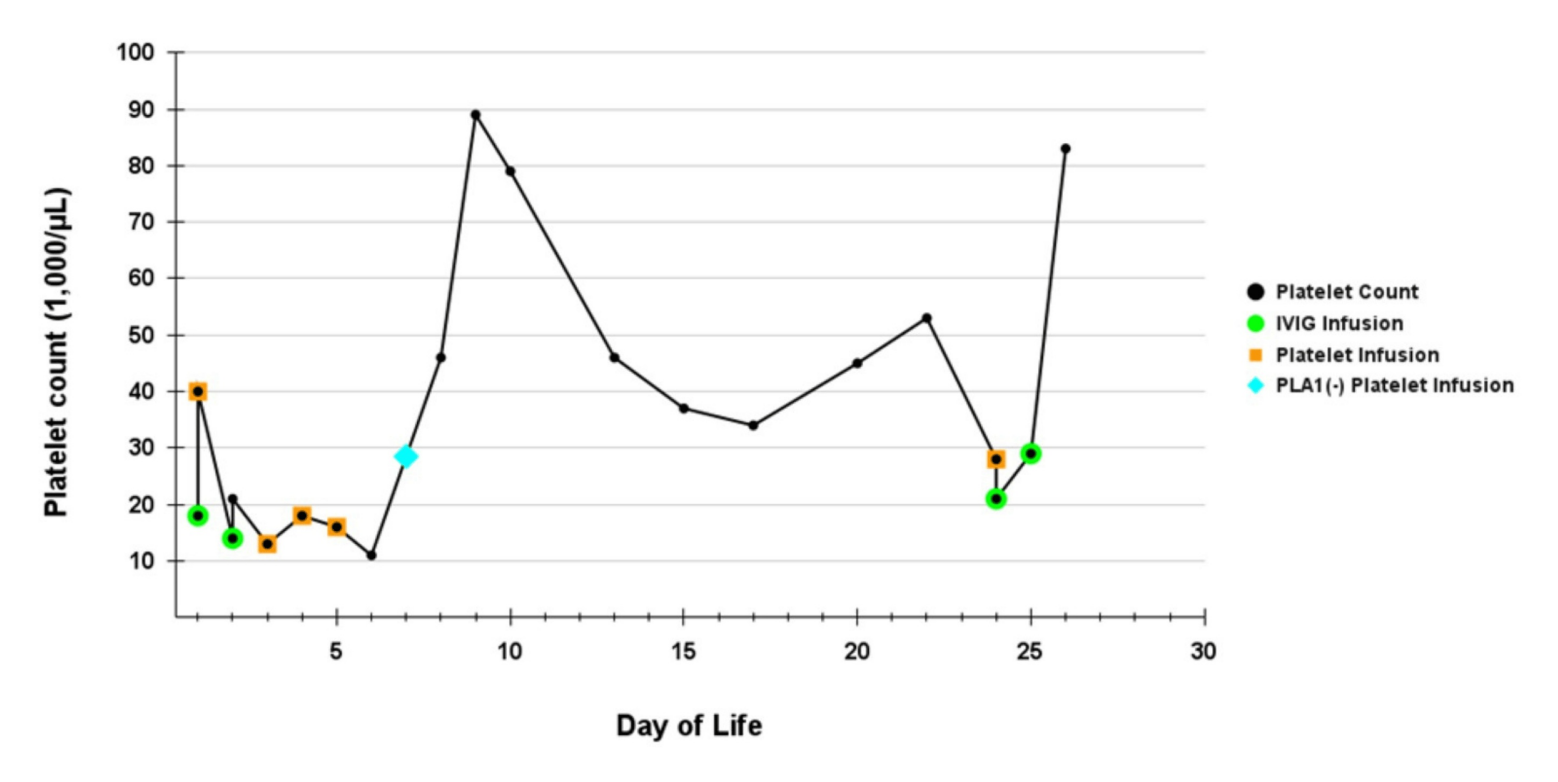
Neonatal platelet counts during NICU admission and timing of platelet and IVIG infusions. The platelet count was 182,000/µL on day of life 29. IVIG: intravenous immunoglobulin

As shown in Figure 2, she therefore required several subsequent platelet transfusions, multiple IVIG courses, and also adjunctive prednisone. PLA1-negative platelets were administered on day 7, with a transient improvement. Serial cranial ultrasounds remained negative for intracranial hemorrhage. At discharge on day 29, she was sent home on a tapering dose of prednisone 3.9 mg (1.1 mg/kg) daily, which represented a taper dose of 6.8 mg (2 mg/kg), and her platelet count had normalized to 182,000/µL. She remained clinically stable and was followed by pediatric hematology, with platelet counts of 51,000/µL and 116,000/µL at one week and one month post-discharge, respectively.

## Discussion

The diagnosis and management of ITP in pregnancy are complex, as they require balancing maternal safety, fetal well-being, and delivery planning. Standard recommendations suggest maintaining platelet counts ≥30 × 10⁹/L throughout pregnancy and ≥50 × 10⁹/L at delivery to minimize maternal hemorrhage risk [4,12]. Corticosteroids and IVIG remain the first-line therapies, with splenectomy reserved for refractory cases [5,13]. However, maternal disease severity is not only relevant to maternal outcomes but also predictive of neonatal risk, as highlighted in this case.

Maternal predictors of neonatal disease

Several maternal factors have been consistently linked to risk for neonatal thrombocytopenia. The history of a previously affected neonate is the strongest predictor, with recurrence rates of severe neonatal thrombocytopenic purpura (NIT) reported as high as 75% [9,10]. Maternal splenectomy prior to pregnancy has also been correlated with increased severity of neonatal disease, likely reflecting refractory maternal disease and heightened immune dysregulation [11]. Severe maternal thrombocytopenia near delivery has been shown in multiple cohort studies to correlate with neonatal thrombocytopenia, although the association is not absolute [14,15]. In our case, the pregnant patient’s history of splenectomy, chronic severe thrombocytopenia, and a previously affected child placed the neonate at particularly high risk, which was borne out by the clinical course.

Neonatal course and therapeutic dilemmas

The neonate in this case presented with profound thrombocytopenia at birth. The initial platelet count at 1.25 hours of age was 10,000/µL, necessitating repeated platelet transfusions, IVIG, and adjunctive steroids. This clinical picture reflects the known heterogeneity in neonatal outcomes: while many infants of patients with ITP have mild or no thrombocytopenia, approximately 10-30% develop severe disease, and 1% are at risk for intracranial hemorrhage [7,8,16].

IVIG is the mainstay of neonatal management, given its ability to block Fc receptors and reduce platelet destruction [1,17]. Platelet transfusions are typically reserved for acute bleeding or platelet counts <20-50 × 10⁹/L, though their efficacy is limited by rapid clearance in the presence of circulating maternal antibodies [18,19]. Steroids are less consistently used but may provide benefit in refractory cases [19]. Resistance to standard therapy in this neonate led to the use of platelet A1-negative (PLA1-negative) platelets and steroid administration, underscoring the therapeutic challenges when standard interventions fail.

## Conclusions

This case illustrates a pregnant woman with chronic severe ITP and prior splenectomy who received aggressive management, including corticosteroids, IVIG, and planned elective cesarean delivery to optimize her platelet count before birth. Despite successfully treating the mother's thrombocytopenia, her newborn developed profound thrombocytopenia immediately after delivery. The infant required 29 days of NICU hospitalization with multiple platelet transfusions, IVIG courses, and corticosteroid treatment. The mother had a previously affected infant with similar neonatal complications.

Current practice lacks standardized, evidence-based guidelines for managing the mother-infant dyad in ITP, with most recommendations extrapolated from other conditions. This case highlights the urgent need for multidisciplinary collaboration among obstetricians, hematologists, and neonatologists, along with risk-stratification tools to guide delivery planning and neonatal surveillance. Future research should focus on identifying better predictive biomarkers beyond simple platelet counts, including maternal antibody specificity and immunogenetic factors, to determine which neonates will develop severe disease.
